# Supplementary material for: Design and evaluation of an MRI-ready, self-propelled needle for prostate interventions
Source: PLoS One. 2022 Sep 7;17(9):e0274063. doi: 10.1371/journal.pone.0274063 (PMC9451087; doi:10.1371/journal.pone.0274063)
Supplement: S1 Appendix — (DOCX) [file pone.0274063.s001.docx]

**S1 Appendix. Support structure design and manufacturing**

A support structure for the Ovipositor MRI-Needle and the target tissue was required to facilitate the evaluation of the Ovipositor MRI-Needle performance inside a preclinical MRI system. The preclinical MRI system used in this study is a preclinical 7-T MRI system (MR Solutions, Guildford, United Kingdom). The support structure should provide a steady base for the Ovipositor MRI-Needle that fits within the restricted space available inside the MRI system. Furthermore, the support structure should allow moving the tissue towards the needle with very low friction. Fig S1-1 shows a simplified illustration of the components that make up the MRI system of Fig 8. The MRI system’s housing contains a cylindrical bore (inner diameter 95 mm). A horizontal, half-round tube (inner radius 45 mm) can be slid into and out of the bore. A cylindrical radiofrequency (RF) coil (inner diameter 65 mm) is placed on top of the half-round tube. The MR image is taken inside the RF coil. During the performance evaluation of the Ovipositor MRI-Needle, we were interested in the position of the needle tip; therefore, the tissue was placed inside the RF coil. The Ovipositor MRI-Needle was attached to the half-round tube while aligning the needle with the tissue.

Fig S1-2 shows our developed support structure that consists of an Ovipositor MRI-Needle holder, tube base 1, tube base 2, and needle guide tube. The PMMA support structure that carries the Ovipositor MRI-Needle, called the Ovipositor MRI-Needle holder (Fig S1-2), was produced using laser cutting. The Ovipositor MRI-Needle holder has a flat base; therefore, a component was required that flattens the half-round tube and that lifts the Ovipositor MRI-Needle such that the needle is centrally aligned in the MRI bore. Tube base 1 and 2 fulfil these functions. They were produced out of PLA using the Ultimaker 3. To prevent buckling of the needle before entering the tissue, the needle was supported by the needle guide tube (Fig S1-2). The needle guide tube is a PLA tube with an inner diameter of 2 mm and an outer diameter of 3 mm. An extension piece was added to the translation ring to allow manual actuation of the Ovipositor MRI-Needle from outside the MRI-bore.

A low-friction structure was required to allow horizontal translation of the tissue inside the RF coil (Fig S1-3). This structure had to constrain the rolling motion in the lateral direction but allow a low-friction rolling motion in the axial direction. The tissue had to be centrally aligned in the RF coil, as this position provides the most space for the tissue and results in the least distortions in the MR image.

In the low friction structure, the tissue was placed in a PLA box (height 23 mm, length 100 mm, width 60 mm, weight 58g) produced with a Prusa 3D printer. The proximal side of the tissue box contained 17 insertion holes aligned on the x-axis at a 2.5-mm centre-to-centre distance. The insertion holes guided the needle into the tissue in the tissue box. Inside the RF coil, a set of guiding rails was placed. These RF base rails provide a stationary horizontal track. On top of the RF base rails, eight wheels (made from LEGO) were placed as rolling elements: four wheels horizontally aligned at the left and four at the right. Spacers aligned the wheels at a 25-mm centre-to-centre distance; wheel axes were not used. Underneath the tissue box, another set of rails was attached. These box rails provided upper rails for the wheels. The wheels rolled at an angle of 45° between the RF base rails and the box rails, which ensured, in combination with the weight of the tissue box, that the wheels were horizontally aligned without using wheel axes.


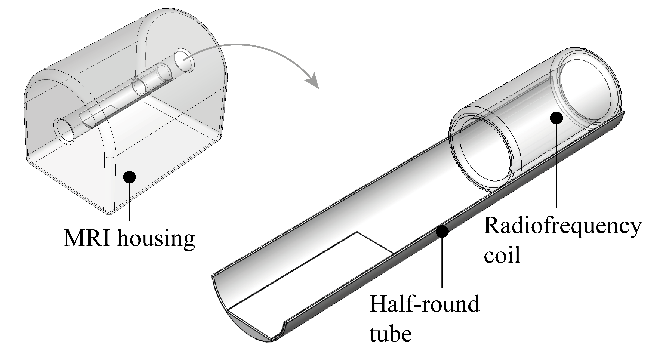


**Fig S1-1.** Illustration of the MRI system used in this study, showing the MRI housing with a half-round tube and a radiofrequency coil that can be slid into and out of the MRI housing.


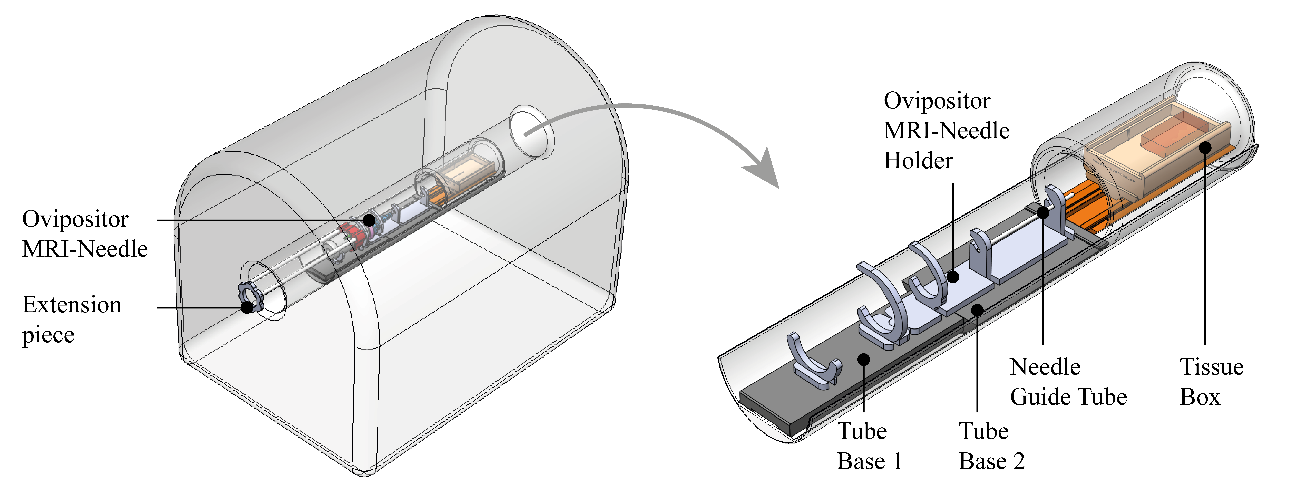


**Fig S1-2.** Illustration of the MRI system used in this study with the support structures to attach the Ovipositor MRI-Needle to the half-round tube and allowing the tissue box to roll inside the radiofrequency coil. The extension piece allows manual actuation of the Ovipositor MRI-Needle from outside the MRI system.

The restricted space inside the RF coil demanded a solution for the rails and wheels that occupied little space while allowing a low-friction rolling motion. Furthermore, the tissue box on wheels had to remain centrally aligned on the rails when the needle was inserted through one of the off-centre insertion holes, resulting in an off-centre axial force that pulls the tissue towards the needle. For wheels placed at a 0°-angle, there exists a trade-off between narrow rails to keep the tissue box on wheels centrally aligned in the RF coil and wide rails to allow the wheels to roll smoothly and prevent jamming of the wheels when pulling the tissue from the off-centre insertion holes. The wheels at a 45°-angle keep the box rails’ motion in the lateral direction aligned with the RF base rails, thereby preventing jamming of the wheels when pulling the tissue from the off-centre insertion holes. Flanges on the RF base rails keep the wheels on the RF base rails, preventing derailing.

**
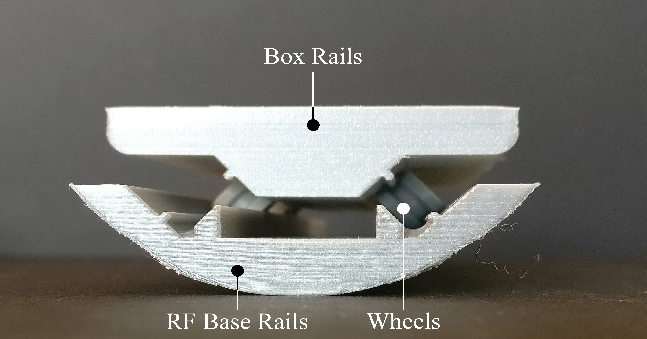
**

**Fig S1-3.** Photograph of rails used to move the tissue sample. The RF base rails fit inside the radiofrequency coil of the MRI system. Wheels between the upper box rails and the lower RF base rails allow sliding of the box rails with respect to the RF base rails.
