# Supplementary material for: Design and evaluation of an MRI-ready, self-propelled needle for prostate interventions
Source: PLoS One. 2022 Sep 7;17(9):e0274063. doi: 10.1371/journal.pone.0274063 (PMC9451087; doi:10.1371/journal.pone.0274063)
Supplement: S2 Appendix — (DOCX) [file pone.0274063.s002.docx]

**S2 Appendix. Magnetic resonance parameters**

This appendix contains the magnetic resonance (MR) parameters set for the *ex vivo* experiment in this study. Table S2-1 shows the MR parameters set for the MR images of the *ex vivo* prostate experiment.

**Table S2-1.** MR parameters for *ex vivo* prostate experiment.

| Parameter | 3D gradient echo |
| --- | --- |
| TR [ms] | 20 |
| TE [ms] | 0 |
| TI [ms] | 1000 |
| Number of averages | 2 |
| Flip angle [°] | 10 |
| Field of view [mm x mm] | 52.00x52.00  x0.29 |
| Matrix size | 192x192 |
| Slice thickness [mm] | 0.29 |
| Slice spacing [mm] | 0.29 |
| Acquisition time [min:s] | 6:09 |

*Abbreviations*. TR = repetition time, TE = echo time, TI = inversion time.
