# Supplementary material for: Design and evaluation of an MRI-ready, self-propelled needle for prostate interventions
Source: PLoS One. 2022 Sep 7;17(9):e0274063. doi: 10.1371/journal.pone.0274063 (PMC9451087; doi:10.1371/journal.pone.0274063)
Supplement: S3 Appendix — (DOCX) [file pone.0274063.s003.docx]

**S3 Appendix. Experimental protocol**

The experimental procedure for each measurement inside the prostate tissue was executed in the following sequence of ten steps:

1. Using a rigid 18-Gauge needle, we made an initial needle track of 40 mm in the agar in front of the prostate tissue to ensure that we could insert the needle straight.
2. We manually inserted the needle through an insertion hole in the tissue box over 40 mm in the initial needle track to allow the self-propelled mechanism to work.
3. We placed the tissue box on the wheels on an RF base plate inside the RF coil.
4. We attached the housing of the Ovipositor MRI-Needle to the half-round tube while horizontally aligning the needle with the tissue box’s insertion hole.
5. To ensure visualisation of the needle, we slid the experimental setup on the half-round tube into the MRI bore.
6. To obtain the needle tip’s start position in an MR image, we made a 3D gradient echo acquisition.
7. We manually actuated the needle for five cycles (60 translations) which corresponds to a theoretical distance travelled of 20 mm.
8. To capture the needle tip position in an MR image, we made a 3D gradient echo acquisition.
9. We manually actuated the Ovipositor MRI-Needle for another five cycles.
10. To capture the needle tip position in an MR image, we made a 3D gradient echo acquisition.
