# Supplementary material for: Design and evaluation of an MRI-ready, self-propelled needle for prostate interventions
Source: PLoS One. 2022 Sep 7;17(9):e0274063. doi: 10.1371/journal.pone.0274063 (PMC9451087; doi:10.1371/journal.pone.0274063)
Supplement: S4 Appendix — (DOCX) [file pone.0274063.s004.docx]

**S4 Appendix. Study data and original MR images**

This appendix contains the original MR images of the *ex vivo* prostate tissue experiment and the measurements performed during the experiment, including the measurements after every set of five cycles. In the “Evaluation” Section, the MR images contain colour indications for the needle, tissue, sides of the box, and the reference point in the box to measure the slip ratio. Fig S4-1 shows the original MR images without the colour indications.

| 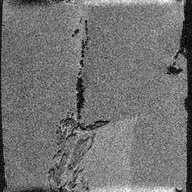  (A) | 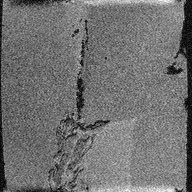  (B) |  |
| --- | --- | --- |
| 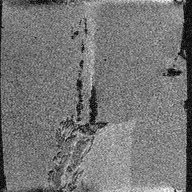  (C) | 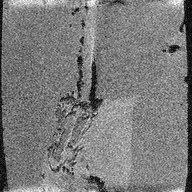  (D) | 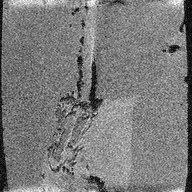  (E) |
| 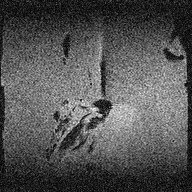  (F) | 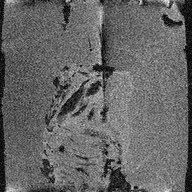  (G) |  |

**Fig S4-1.** MR images of the needle inside *ex vivo* prostate tissue embedded in agar. Each row represents one measurement number. The first column shows the initial frame where the tip is positioned inside the agar in front of the prostate tissue. The second column shows the frame after actuation for five cycles. The third column shows the frame after the second actuation for five cycles. The figure continues on the next page.

| 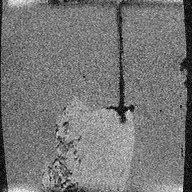  (H) | 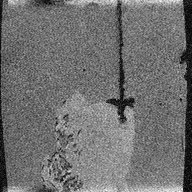  (I) | 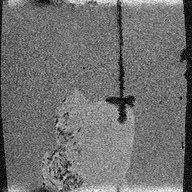  (J) |
| --- | --- | --- |
| 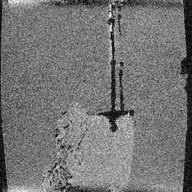  (K) | 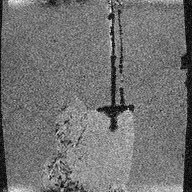  (L) | 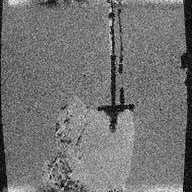  (M) |

**Fig S4-1.** MR images of the needle inside the agar and *ex vivo* prostate tissue. Each row represents one measurement number. The first column shows the initial frame where the tip is positioned inside the agar in front of the prostate tissue. The second column shows the frame after actuation for five cycles. The third column shows the frame after the second actuation for five cycles.

Table S4-1 shows the results of the *ex vivo* evaluation, including the results after every set of five cycles of actuation. The results from measurement numbers 4 and 5 were removed because the needle moved below or over the tissue instead of through the tissue.

**Table S4-1.** Results of the *ex vivo* evaluation. For each measurement, the following information is reported: the insertion hole used on the box, where hole 9 is the centre hole, travelled distance [mm] of the box, number of cycles needed to travel that distance, theoretical travelled distance [mm] that the box would have travelled if no slip occurred, and slip_ratio_. The travelled distance, theoretical travelled distance and slip_ratio_ are shown for the entire measurement and for each set of five cycles.

| Measurement number | Insertion hole | Initial insertion depth [mm] | Travelled distance [mm] of the box | Cycles | Theoretical travelled distance [mm] | slip_ratio_ |
| --- | --- | --- | --- | --- | --- | --- |
| 4 (removed) | 10 | 43.9 | 0 | 5 | 20 | 1.0 |
| 1 | 9 | 44.1 | 8.8  7.0  1.8 | 10  1^st^ 5 cycles  2^nd^ 5 cycles | 40  20  20 | 0.78  0.65  0.91 |
| 5 (removed) | 8 | 49.0 | 9.7 | 5 | 20 | 0.51 |
| 2 | 6 | 50.9 | 2.3  1.8  0.5 | 10  1^st^ 5 cycles  2^nd^ 5 cycles | 40  20  20 | 0.94  0.91  0.97 |
| 3 | 7 | 51.9 | 1.9  1.7  0.2 | 10  1^st^ 5 cycles  2^nd^ 5 cycles | 40  20  20 | 0.95  0.92  0.99 |
